# Awareness and compliance of dental students and interns toward infection control at Riyadh Elm University

**DOI:** 10.3205/dgkh000326

**Published:** 2019-08-05

**Authors:** Sultan Binalrimal, Abdulmajed AlDrees, Mohammed AlWehaibi, Mohammed AlAsmary, Abdulaziz AlShammery, Essam AlHaidri, Lama AlQabbaa

**Affiliations:** 1 Restorative Dentistry Department, Riyadh Elm University, Riyadh, Saudi Arabia; 2 Riyadh, Saudi Arabia; 3 Prosthodontics Department, King Salman Armed Forces Hospital – North Western Region, Tabuk, Saudi Arabia; 4 Public Health Department, Riyadh Elm University, Riyadh, Saudi Arabia; 5 Ministry of Health, Riyadh, Saudi Arabia; 6 Riyadh Elm University, Riyadh, Saudi Arabia

**Keywords:** cross-infection, infection control, dental students, dental interns

## Abstract

**Aim:** Dental students have increasing patient contact during their education and clinical years, putting them at high risk of cross-infection; therefore, the purpose of this study was to determine the level of infection control practices among dental students and interns at Riyadh Elm University, Riyadh, Saudi Arabia.

**Methods:** Total of 400 questionnaires were distributed among interns and clinical students at Riyadh Elm University. The questionnaire comprised 32 items assessing infection control practices, and the data were tabulated and analyzed by SPSS to produce descriptive statistics.

**Results:** Three hundred nine questionnaires were answered (response rate 77%).The implementation of different infection control measures was good to excellent, except for hepatitis B vaccination and wearing eye protection: only 76% of males and 83% of females were vaccinated against HBV, and only 30% of males and 26% of females always wore protective glasses.

**Conclusion:** Compared to previous studies, an increased awareness regarding infection control practices among dental students and interns was noticeable. However, greater emphasis on the importance of infection control, especially compliance with HBV vaccination and wearing protective eyewear, is necessary.

## Zusammenfassung

**Zielsetzung:** Zahnmedizinstudenten und Praktikanten sind während ihrer klinischen Ausbildung Infektionsrisiken ausgesetzt. Daher sollte die Einhaltung der Schutzmaßnahmen der Auszubildenden an der Riyadh Elm University, Saudi Arabien, analysiert werden.

**Methode:** Es wurden 400 Fragebögen mit 32 Items zur Einhaltung ausgewählter Präventionsmaßnahmen ausgegeben. Die Daten wurden mittels SPSS deskriptiv ausgewertet.

**Ergebnisse:** 309 Fragebögen waren auswertbar (Response Rate 77%). Die Implementierung der Präventionsmaßnahmen war mit Ausnahme der HBV-Schutzimpfung und des Tragens einer Schutzbrille gut bis sehr gut. Geimpft waren jedoch nur 76% der männlichen and 83% der weiblichen Personen. Augenschutz trugen sogar nur 30% der männlichen and 26% der weiblichen Personen.

**Schlussfolgerung:** Im Vergleich zu vorangegangen Analysen wurde ein gesteigertes Bewusstsein für Infektionskontrollpraktiken bei zahnmedizinischen Studenten und Praktikanten festgestellt. Es ist jedoch notwendig, dass alle Auszubildenden zur HBV-Schutzimpfung und zum Tragen von Schutzbrillen motiviert werden.

## Introduction

Dental health care workers (DHCW) are at a high risk of cross-infection through occupational exposure, such as needlestick and sharp instrument injuries (NSIs), mucocutaneous contamination, bites, conjuctivitis and mechanical trauma. The eyes are at particular risk from floating particles, which are considered serious hazards for DHCW, because they can transmit various microorganisms, e.g., cytomegalovirus, *Mycobacterium tuberculosis*, hepatitis B and hepatitis C viruses (HBV and HCV), *Herpes simplex* virus type 1, human immunodeficiency virus (HIV), streptococci, transmissible spongiform encephalopathies (including variant CJD), methicillin-resistant *Staphylococcus aureus* (MRSA), and severe acute respiratory syndrome (SARS) virus transmitted through direct and indirect contact [1], [2], [3], [4], [5], [6], [7], [8], [9], [10], [11], [12], [13], [14], [15], [16], [17].

The majority of infections are subclinical and almost 80% of all HBV infections are undiagnosed. Therefore, DHCW are at risk every day, because at normal working distances, there is no zone of safety for the risk of cross-infection. The danger is enhanced by the fact that some hazardous microorganisms remain in the air for up to 30 minutes after cavity excavation, and the fact that most human microbial pathogens have been isolated from oral secretions [7], [8], [18], [19], [20].

In 1946, Humphrey [21] described how three dental staff members developed different infections including syphilis, diphtheria and actinomycosis during the treatment of infected patients. The protection of health workers became imperative, especially with the rise of of the AIDS epidemic in the 1980s [22].

Routine infection control procedures and recommendations have been available since the 1970s, but were neglected and ignored even by highly educated groups. In 1996, the US Centers for Disease Control and Prevention (CDC) adopted the term “standard precautions” to generate broader understanding and awareness of prevention and transmission of infection. In 2003, the CDC published “Guidelines for Infection Control in Dental Health-Care Settings”, which included standard precautions to ensure safe working environments and prevent cross-infection among DHCW and their patients, and in 2016 the CDC published “Summary of Infection Prevention Practices in Dental Settings: Basic Expectations for Safe Care” [22], [23], [24], [25].

Despite the emphasis that was placed on making DHCW aware of the risk of cross-infection and the recommended practices to control infection transmission, the percentage of DHCW who adhered to those practices was below expectations [1], [2], [4], [9], [26], [27], [28], [29], [30], [31], [32], [33], [34], [35], [36], [37], [38].

Even at dental schools, dental students did not comply with infection control practices very well [13], [31], [34], [39], [40], [41], [42], [43], [44], [45], [46], [47], [48], [49], [50], [51]. 

Because dental students have increasing patient contact during their education and clinical years, they are at massive risk of cross-infection. Therefore, the aim of this study was to determine the level of compliance with infection control practices among dental students and interns at Riyadh Elm University, Riyadh, Saudi Arabia.

## Materials and methods

The study was approved by the Review Board and Ethics Committee of Riyadh Elm University. A total of 400 questionnaires were distributed among clinical students and interns at Riyadh Elm University, Riyadh, Saudi Arabia. The questionnaire was designed to cover different aspects of infection control practices in dental clinics including:

1. aseptic techniques,
2. patient screening and evaluation,
3. personal protection,
4. instrument sterilization,
5. immunization against hepatitis B virus,
6. surface disinfection, and
7. equipment asepsis.

An informed consent was included in the questionnaire design and required to be signed by every participant. The questionnaire comprised 32 items assessing infection control practices among undergraduate dental students and interns at Riyadh Elm University. Out of 400 questionnaires sent, 309 forms were filled out and returned. Forty-two of the respondents were interns (21 female and 21 male), while 267 forms were received from both male and female clinical students (96 4^th^-year students, 85 5^th^-year students, 86 6^th^-year students). The results obtained from respondents were tabulated and analyzed using the Statistical Package for the Social Sciences, version 24 (SPSS Inc. Chicago, IL USA).

## Results

309 questionnaires were answered by clinical dental students and interns from Riyadh Elm University. The distribution of respondents from male and female 4^th^-, 5^th^-, and 6^th^-year students and interns is shown in Table 1 (Tab. 1).

The level of implementation of basic applied infection control measures was found to be good to excellent, except for HBV vaccine coverage and wearing protective eyewear. The results showed that only 76% of males and 83% of females had HBV vaccination, and only 30% of males and 26% of females always wore protective eyewear (Table 2 (Tab. 2) and Table 3 (Tab. 3)).

A majority of dental students and interns complied well with different infection control measures during the treatment of patients and between patients, but there is still a need to raise their awareness and improve their attitude regarding infection control practices. The infection control practices followed by dental students and interns during the treatment of patients and between patients are shown in Table 4 (Tab. 4).

## Discussion

Infection control is a dynamic and ever-changing discipline. Standard isolation precautions are designed to reduce the risk of acquiring occupational infections from both known and unknown sources in healthcare settings. Awareness of and compliance with these recommendations are crucial for the prevention of infections among healthcare workers (HCW), including dental healthcare professionals. The recrudescence of diseases such as HIV, tuberculosis, hepatitis B and C, and other maladies that were on the decline have made it essential that strict sterilization be maintained. The results of this study showed that the implementation of different infection control measures was good to excellent, except for hepatitis B vaccination and wearing eye protection.

Saudi Arabia was one of the HBV-endemic countries with an overall prevalence of 8.3%. The infection was mostly spread through horizontal transmission in early life. Over the years, there has been an enormous decrease in HBV incidence in Saudi Arabia, and the prevalence rate in 2009 was found to be 1.31% which places Saudi Arabia among the countries with the lowest prevalence rate of HBV worldwide [12], [13], [14], [15], [16], [17]. Unfortunately, previous studies reported that HCW in Saudi Arabia showed low compliance with hepatitis B vaccination, and it became mandatory to structure educational programs to raise the awareness and enhance health-care workers’ compliance with HBV vaccination [26], [37], [38]. It is noteworthy that HBV vaccination compliance was also below expectations among HCW in Brazil, Serbia, Libya, Ethiopia, China and India [10], [11], [25], [26], [27], [28], [35], [36], [42].

In previous studies investigating dental students’ and interns’ knowledge of, attitude toward and compliance with HBV vaccination among private and governmental dental schools in Saudi Arabia, their attitude was positive regarding infection control protocols, but they unfortunately lacked compliance with HBV vaccination; greater emphasis on the importance of immunization and vaccine coverage was needed. In our study population the compliance was greater than that of these other studies [33], [37], [45], [46], [47], [48], [49], [50] but still in need of improvement. 

The results of this study demonstrate a need to further emphasize eye protection. The importance of protective eyewear was found to be in the interest of both dentist and patient due to the hazards associated with aerosols and floating debris. The use of eye protections with side shields, and regular monitoring of its structural integrity, reduces the risk of conjunctivitis, eye damage or even complete loss of vision [8].

The use of protective eyewear by dental students, interns and dentists in Saudi Arabia was found to be low in previous studies [33], [37], [45], [46], [47], [48], [49], [50]. In our study population the compliance was greater, but still in need of improvement. 

In addition, the low compliance with eye protection was reported among DHCW and students in Lebanon, Morocco, India, and China [30], [35], [36], [40], [42].

It is the ethical obligation of every health care worker to safeguard themselves and their patients from cross-infection. Because the community expects zero risk of infection transmission from health care providers, a novel, more effective approach is needed to raise the awareness of the importance of vaccine coverage, protective eyewear, and adherence to infection control protocols.

## Conclusion

Dental students tend to practice infection control measures they acquired during their clinical practice, and they are the future dental professionals who will provide oral healthcare to the population. Therefore, it is the responsibility of every academic institution to facilitate an appropriate preclinical immunization program, provide infection control training to protect students and patients from cross-infection, and educate the future healthcare professionals about safe work practices. The results of this study indicated increased awareness among concerned dental students and interns at Riyadh Elm University towards the implementation of effective infection control measures. Despite the increase awareness, more emphasis on the importance of compliance with HBV vaccination and the adherence to protective eyewear is needed.

## Notes

### Competing interests

The authors declare that they have no competing interests.

## Figures and Tables

**Table 1:**
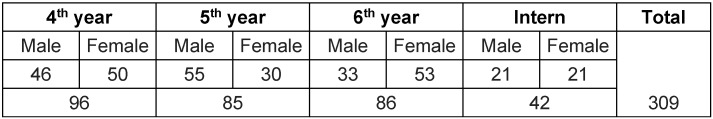
Distribution of respondents in the college to 4^th^-, 5^th^-, and 6^th^-year students and interns both male and female

**Table 2:**
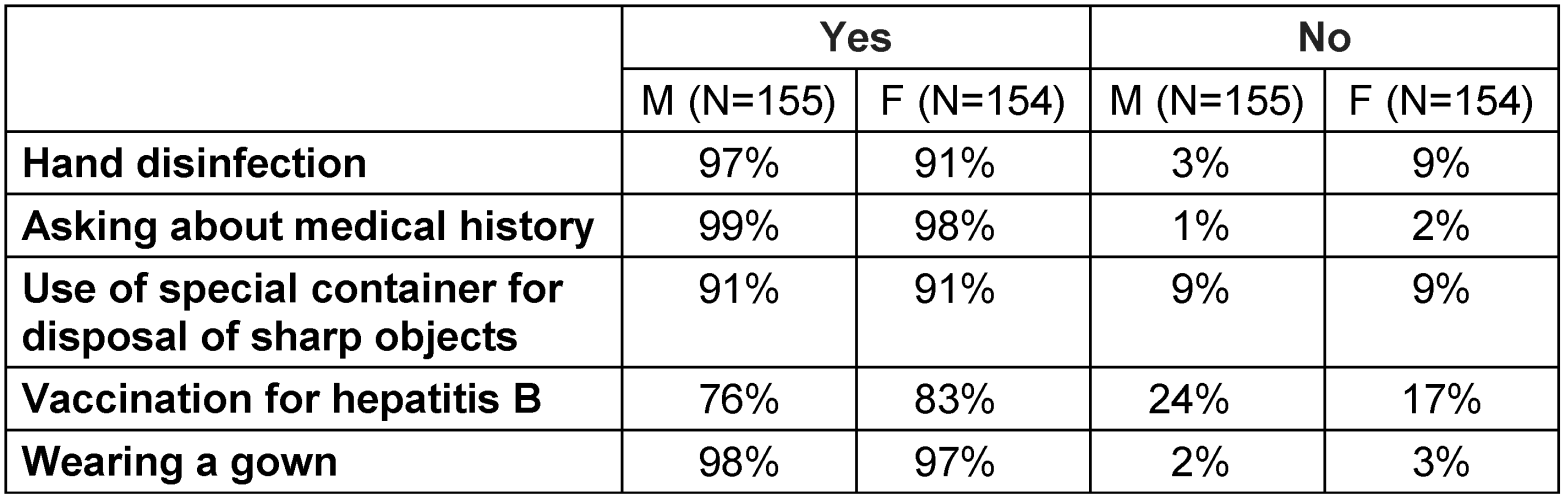
Level of implementation of basic infection control measures at the university dental school

**Table 3:**
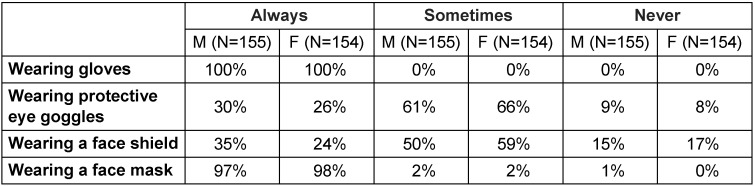
Table 3 : Level of implementation of basic infection control measures at the university dental school

**Table 4:**
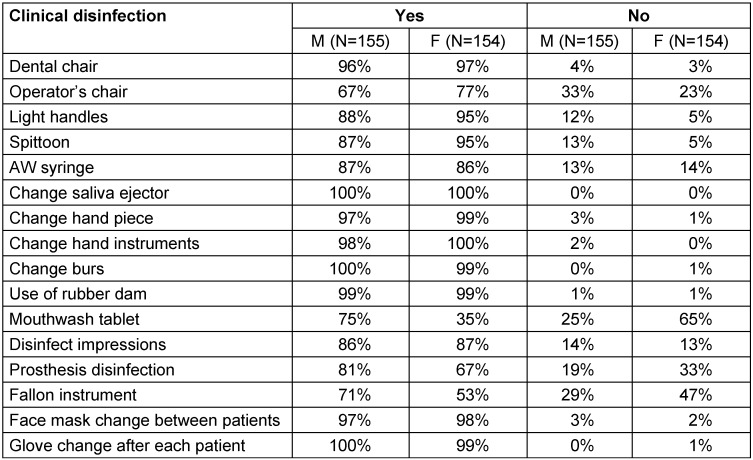
Infection control measures followed during treatment and between patients in the clinic
